# Green and scalable surface functionalization of silicon quantum dots using water-soluble organic acids for sustainable hybrid materials

**DOI:** 10.1039/d5ra08090h

**Published:** 2026-01-14

**Authors:** Galář Pavel, Čandová Gabriela, Matějka Filip, Hassouna Fatima, Laachachi Abdelghani, Sajdl Petr, Kůsová Kateřina

**Affiliations:** a Institute of Physics of the CAS v.v.i., Cukrovarnická 10 162 00 Prague 6 Czechia galar@fzu.czac; b Faculty of Chemical Engineering, University of Chemistry and Technology, Prague Technická 5 166 28 Prague 6 Czechia; c Luxembourg Institute of Science and Technology (LIST), Maison de l'innovation 5 Avenue des Hauts-Fourneaux Esch-sur-Alzette Luxembourg; d Faculty of Environmental Technology, University of Chemistry and Technology Technická 5 166 28 Praha 6 Czechia

## Abstract

Silicon quantum dots (SiQDs) are attractive components for hybrid materials due to their inherently low toxicity, tunable optical properties, high lithium-specific capacity, and mechanical resilience. However, their integration is often hindered by the challenge of forming stable aqueous dispersions, due to their hydrophobic character and susceptibility to oxidation. In this work, we demonstrate that two common and environmentally benign acids, poly(acrylic acid) and phytic acid, can serve not only as dispersants but also as effective surface termination agents for SiQDs. In particular, the modification of surface hydrides was shown to lead to mixed covalent (Si–O–C, Si–O–P) and ionic bonding, resulting in enhanced colloidal stability. The robust covalent attachment and electrostatic stabilization by deprotonated acid groups was observed to effectively shield the terminated SiQDs from the highly reactive low-pH water. The shielding was monitored by photoluminescence measurements when initially H-terminated SiQDs showed minimal photoluminescence quenching at pH as low as 1.5 after treatment with phytic acid, compared to a 50% photoluminescence loss in untreated SiQDs. These beneficial effects were significantly diminished when mildly oxidized SiQDs underwent the treatment as a result of the lower availability of the reactive Si–H surface groups, or when both the acids were combined. Our findings show that the observed benefits arise from the reactive hydrogenated surface, often lacking in applications like Li-ion batteries and hybrid supercapacitors. Thus, this work highlights a green strategy for utilizing hydrogen-terminated SiQDs synthesized by non-thermal plasma, followed by benign acid-based functionalization in water, enabling nanohybrid synthesis without hazardous chemicals.

## Abstract 2

Hydrogen-terminated SiQDs are passivated by phytic acid (PhA) using a simple, low-cost, green method. Coulombic fields from PhA provide exceptional stability even at very low pH, preserving optical properties for sensing and bio-applications.

## Graphical abstract

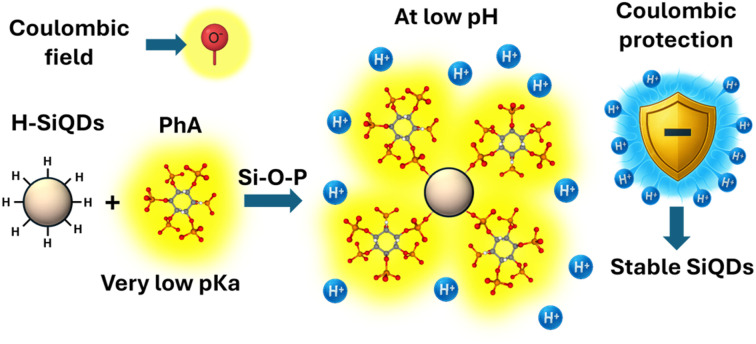

## 1. Introduction

Silicon quantum dots (SiQDs) have attracted considerable interest, owing to appealing properties combining inherently low toxicity and general abundance of this element with tuneable electronic properties, high surface area, and chemical versatility. Therefore, they are considered across a range of potential technological applications including photonics, bioimaging, photovoltaics, optoelectronics, and energy storage.^1–3^ Moreover, their nanoscale dimensions can mitigate mechanical stress and enhance structural stability in applications where bulk Si suffers from severe degradation, such as lithium-ion batteries or flexible electronics.^4,5^ A key challenge in realizing the potential of SiQDs lies in the development of reliable, scalable, and environmentally friendly surface treatment methods that preserve their unique properties while enabling integration into functional materials and devices.

One promising approach is the use of aqueous processing routes, which are attractive for both economic and environmental reasons. However, achieving a stable dispersion of SiQDs in aqueous media is challenging due to their inherent hydrophobicity after synthesis (related to hydrogen surface termination),^6^ and their strong tendency to oxidize and aggregate, driven by van der Waals forces and inter-particle interactions. Surface modification is therefore essential,^1,7^ not only to improve colloidal stability, but also to tailor the surface chemistry for compatibility with a range of matrix materials, such as polymers or composites. Ideally, this modification should be achieved using green, biocompatible agents under mild processing conditions.

Acidic additives are widely employed in water-based processing of Si-based nanomaterials, where they can serve multiple roles depending on their chemical structure. In particular, poly(acrylic acid) (PAA), a weak polyelectrolyte with abundant carboxylic acid groups, is frequently used as a physical binder^8–10^ and dispersing agent.^11–14^ Its ability to interact with oxidized Si surfaces through hydrogen bonding and electrostatic interactions^9,15,16^ enables improved particle dispersion and enhances mechanical cohesion in composite materials. As another example, phytic acid (PhA), a naturally occurring inositol hexaphosphate, is widely employed as a multivalent cross-linker and surface-modifying agent, particularly in the synthesis of conductive polymer networks such as polyaniline and polypyrrole PPy.^16–19^ Through multivalent interactions, PhA can promote inter-particle cross-linking, modify surface charge, and influence the assembly of nanostructures. While neither PAA nor PhA is traditionally classified as a surface functionalization agent, both can significantly alter the surface properties of SiQDs and their behaviour in aqueous suspension. Thus, despite the interest in both PAA and PhA, motivated mostly by the fabrication of Si-based anodes in aqueous media for Li-ion batteries and hybrid supercapacitors, either as additives, or as conductive binders, their broader potential in green, water-based processing of Si nanomaterials remains underexplored. Specifically, the fundamental interactions between these acids and SiQDs surfaces remain insufficiently understood, particularly in aqueous environments where surface chemistry is dynamic and often poorly controlled.

The insufficient understanding is further complicated by the fact that as-synthesized SiQDs (typically hydrogen-terminated) are highly susceptible to surface oxidation upon exposure to water or oxygen, leading to the formation of Si–OH or Si–O–Si groups that dramatically alter surface reactivity and cause agglomeration through surface oxygen bridging.^1,2^ In real-world applications, such as aqueous electrode processing, perfect atmospheric control is rarely maintained, and varying degrees of surface oxidation can be expected. Despite the potentially high influence on the stage of surface oxidation of the initial SiQDs material, this important parameter is rarely reported or discussed.

To address these knowledge gaps, we introduce a wet-chemistry procedure for the termination of SiQDs in aqueous media using environmentally benign PhA and PAA acids. To keep the procedure simple and compatible with the anode fabrication process, no atmospheric control is employed. We use SiQDs samples synthesized in non-thermal plasma, which yields highly crystalline, size-controlled nanoparticles with hydrogen surface passivation. To reveal the influence of the initial stage of oxidation on the progression of the surface treatment, we compared freshly synthesized hydrogen-terminated SiQDs (H-SiQDs) with minimal exposure to air and partially oxidized SiQDs (pO-SiQDs), obtained by controlled air exposure, as the starting material for the surface treatment. While the partially oxidized SiQDs are similar to Si nanomaterials conventionally employed in the fabrication of Si anodes, H-SiQDs represent a non-traditional, more reactive platform for the surface functionalization process. Our combined analysis of Fourier Transform Infrared Spectroscopy (FTIR) and X-ray Photoelectron Spectroscopy (XPS) shows that the treatment procedure leads to the formation of stable Si–O–C or Si–O–P linkages, respectively, implying that PhA and PAA can serve as functionalization agents for SiQDs. The functionalization of SiQDs improved dispersibility in aqueous media, reaching sub-100 nm agglomerates over a wide range of pH values. Moreover, the functionalization suppressed low-pH-induced PL quenching and agglomeration, which signifies that the functionalizing agents can effectively shield the surface of the QDs from the highly reactive low-pH water. The mechanism of stabilization was explained using the neutralization and deprotonation curves of the QDs and the functionalizing agents. Even though the beneficial effects of the surface passivation treatment occurred for both types of samples, the best results were obtained with PhA-treated H-SiQDs because of a combination of two factors: the high resistance of PhA against dissociation and the more reactive Si–H surface, which causes a more effective attachment of PhA molecules. Beyond these performance advantages, our approach aligns with green chemistry principles by eliminating hazardous reagents such as hydrofluoric acid, avoiding toxic or solvent-intensive termination procedures, and enabling water-based processing compatible with Li-ion battery fabrication. Thus, we show that the hydrogenation of Si surfaces can serve as a simple tuning parameter in the aqueous processing of Si-based nanomaterials.

## 2. Experimental section

### 2.1 Materials

The SiQDs were synthesized through non-thermal plasma technique. Within this method we applied following working/precursor gasses: silane diluted in argon (1% SiH_4_ in Ar, Linde; Ar 5.6, SiH_4_ 5.0) and hydrogen (H_2_ 7.0, Linde). The termination technique used two acids, in particular poly(acrylic acid) (PAA, *M*_w_ = 450 000) from Merck and phytic acid solution (PhA, 50% (w/w) in H_2_O, *M*_w_ = 660.04) from Merck.

### 2.2 Synthesis of H-SiQDs and preparation of pO-SiQDs

H-SiQDs were synthesized using a custom-made, non-commercial non-thermal plasma flow-through reactor operating in a low-pressure, as described in detail elsewhere.^20^ Briefly, the method is based on the reaction of the working gas in plasma reactor. In the reactor, Si–H bonds in silane molecules are dissociated, followed by the formation of Si–Si bonds, which leads to the gradual growth of silicon seeds. These seeds subsequently coalesce and crystallize upon further heating, mainly as a result of collisions with argon atoms. This process is often referred to as silicon polymerization due to its analogy with the formation of conventional carbon-based polymers. Size of the resulting SiQDs is affected by silane concentration, used plasma power and also concentration of added hydrogen as was demonstrated elsewhere.^20^ Used system is inspired by the design presented by Kortshagen *et al.*^21,22^ It consists of a glass tube reactor equipped with copper plate electrodes (5 cm × 12 cm), and a radiofrequency power source (Coaxial Power Systems, 0–600 W) operating at 13.56 MHz. The reactor is connected to a collecting chamber separated by a slit and evacuated using a dry scroll pump (Edwards XDS35i). H-SiQDs were synthesized with flow rates of 80 SCCM 1% SiH_4_ mixture in Ar and 10 SCCM of H_2_ at 150 W. The system pressure was approximately 10 Pa in standby mode and remained below 500 Pa during the synthesis, corresponding to the residence time of approximately 23 ms. Synthesis durations were 3 minutes for samples yielding 2.5 mg and 10 minutes for those yielding 10 mg of H-SiQDs. The conversion rate is about 80%. To prevent exposure to ambient atmosphere, the as-prepared H-SiQDs were sealed under a nitrogen atmosphere within the collecting chamber (air exposition was about 5 min). The pO-QDs were obtained by exposing H-SiQDs to atmospheric air under laboratory conditions (uncontrolled humidity ranging 55–75%) for one week.

### 2.3 Modification procedure of the SiQDs

For PAA treatment of H-SiQDs, the Si powder was first homogenized in a mortar for 10 minutes, followed by the addition of PAA and mixing for another 10 minutes. The resulting powder mixture was then dispersed in deionized water using a sonication bath. This produced a 0.14 wt% PAA dispersion corresponding to a Si : PAA mass ratio of 1 : 3.6 following the stabilization approach reported by Park *et al.*^23^ The dispersion was stirred magnetically for 1 hour (pH of the slurry was ∼5), centrifuged, and the precipitate containing the SiQDs was collected and dried. The PhA treatment followed a similar protocol (except for the pH of the slurry was ∼3). The combined treatment incorporated all steps from the previous methods: Si powder was homogenized in a mortar (10 minutes), mixed with PAA (10 minutes), dispersed in DI water with PhA using sonication, followed by magnetic stirring for 1 hour (pH of the slurry was ∼3), centrifugation, and drying. In this case, PAA and PhA solutions were mixed at a 1 : 1 ratio, corresponding to an overall ratio of Si : PAA : PhA = 1 : 3.6 : 3.6. The pH of the control solution containing only H-SiQDs in deionized water was measured, and it corresponded to the pH of the water itself (≈6).

### 2.4 Structural and surface characterization

The structure and size of SiQDs were characterized using High resolution transient emission microscope (HRTEM) Joel 2200 FS using a ZrO/W FEG operated at 200 kV. HRTEM samples were prepared by drop-casting a toluene solution containing the SiQDs onto carbon-coated copper grids. The data from the HRTEM were analyzed manually using the ImageJ software (we verified that the extracted histograms are independent of the person carrying out the analysis). The Fourier-transform infrared spectroscopical analysis was carried out using Nicolet (Thermo Scientific) FTIR spectrometers, models iS50 and iS50 ABX. The measurements were performed using attenuated total reflectance (ATR) with a monocrystalline diamond crystal within the spectral range from 400 to 4000 cm^−1^. The signal was collected over 256 scans with a resolution of 2 cm^−1^. OMNIC software was used for data acquisition, baseline subtraction, and signal processing. The elemental analysis and surface chemistry were also tested using two different X-ray photoelectron spectroscopy systems. Namely, Omicron Nanotechnology's ESCA. Probe instrument was used, which uses a monochromatic X-ray source with an energy of 1486.7 eV. The measurements were performed in the CAE (Constant Analyzer Energy) mode with a pass energy of 50 eV for survey spectra and 30 eV for details. A low-energy electron source with energies up to 3 eV was used for possible charge compensation. The spectra were evaluated in CasaXPS, using a function determined from the copper line analysis to calibrate the intensities. The second used system was Axis Ultra DLD from Kratos Analytical Ltd. Hemispherical electron analyzer using a monochromate Al Kα source (1486.6 eV), at a base pressure of 1 × 10^−9^ mbar and power of 60 W. Photoelectron emission take-off angle of 0°; Thermofisher Nexsa G2 and binding energies were calibrated using the main C 1s peak at 284.0 eV as internal standard.

### 2.5 Optical characterization and analysis of the dispersion

The steady-state photoluminescence (PL) spectra of the SiQDs were obtained using the imaging spectrograph Shamrock 300i (Andor, Oxford Instruments) coupled with an EMCCD camera (Newton 971, Andor, Oxford Instruments). The PL spectra of the SiQDs DI water dispersions were measured in fluorescence quartz cuvettes (Hellma, standard cells 10 × 10 mm, 1.0 mL) and excited at 325 nm of intensity 2.5 mW using a continuous wave HeCd laser. The pH was adjusted by adding diluted NaOH and HCl of p.a. quality (P-LAB a.s). All spectra are measured at room temperature and corrected for the response of the whole detection system (using Ocean Optics HL3 Plus calibration lamp). The distribution of the hydrodynamic radius and zeta potential of the objects in the dispersions were characterized using the Zetasizer Nano ZS (Malvern Panalytical) dynamic light scattering (DLS) apparatus equipped with a HeNe laser (633 nm) at scattering angle of 173^°^. Both measurements were performed consecutively under constant pH conditions within folded capillary Zeta Cell (DTS1070, Malvern Panalytical). The dispersions were filtered before measurements using 450 nm PTFE syringe filters (diameter 25 mm, FiltraTECH) to remove objects that would camouflage signal of nanosized objects. Every sample was analyzed 3 times for 50 runs (duration of one run: 10 s). The number-weighted sizes were acquired using the fitting of the autocorrelation function by the built-in software running in the size measuring option. For the calculations we used following material parameters: the refractive index of SiQDs (3.50) and DI water (1.33), as well as the viscosity of the DI water were used 0.89 mPa s at 298.15 K, and 1.002 mPa s at 293.15 K.

## 3. Results

### 3.1 Sample synthesis and basic characteristics

**Fig. 1:**
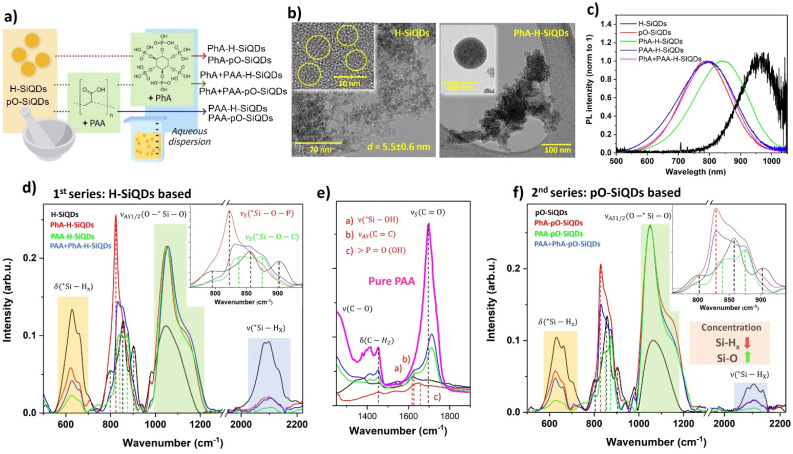
(a) Schematic representation of surface functionalization of SiQDs. (b) TEM images of H-SiQDs (left) and H-SiQDs functionalized by PhA (right). Insets display a magnified view of individual SiQDs and a cluster of SiQDs embedded in excess PhA prior to its removal, respectively. (c) Normalized PL spectra of samples from the H-SiQDs series are compared to pO-SiQDs. (d) FTIR spectra of the H-SiQDs samples. (e) Zoomed-in FTIR spectra of the samples shown in panel (d), focused on the 1250–1900 cm^−1^ region. (f) FTIR spectra of samples from the pO-SiQDs series within a selected interval similar to those in panel (d).

In this study, we used SiQDs synthesized by the non-thermal plasma method, which allows continuous size tuning while attaining a high degree of crystallinity and produces SiQDs with hydrogen surface termination, see the Experimental section.^20^ Notably, hydrogen termination on H-SiQDs is very unstable and prone to oxidation in the presence of water or oxygen.^24,25^ In spite of that, the stage of oxidation of SiQDs undergoing a surface treatment is an often-neglected parameter. For example, the preparation of Li-ion anodes is typically not carried out under an inert atmosphere and, consequently, the interaction with acid and water leads to partial oxidation, while the initial stage of oxidation is typically unreported. Moreover, also most commercial Si nanocrystals exhibit an oxidized surface. Therefore, our goal was to systematically explore the effects of PhA and PAA treatment on SiQDs, and, at the same time, to evaluate the influence of the stage of oxidation of the initial material on the outcome of the procedure. We used two series of samples. The first series (labelled as H-SiQDs) represents surface-treated SiQDs with minimally oxidized surface prior to the surface treatment. It includes (Fig. 1a): (i) the as-synthesized H-SiQDs (crystalline, core size 5.5 ± 0.6 nm, Fig. 1b, left);^20,22^ H-SiQDs treated with (ii) polyacrylic acid (PAA) and (iii) phytic acid (PhA) (Fig. 1b, right), respectively; and (iv) H-SiQDs concurrently exposed to both acids. To examine the effect of surface Si–O bonds on the treatment with acids, a second series was introduced, using moderately oxidized H-SiQDs (labelled as pO-SiQDs) as the starting material for further termination. SiQDs were exposed to ambient air for seven days, which typically results in the formation of a primary stable oxide layer. The second series of samples underwent the same surface treatment procedure, namely, the SiQDs were dispersed in DI water containing an acid-based dispersing agent (*i.e.*, PAA or PhA) using a sonication bath, followed by magnetic stirring. Treated SiQDs were subsequently washed and collected *via* centrifugation. The whole surface-treatment process of SiQDs was carried out in a normal atmosphere, similarly to procedures typical for the fabrication of anodes for Li-ion batteries.^26^

The structural properties of H-SiQD samples were characterized by transmission electron spectroscopy (TEM), confirming the presence of highly crystalline cores in the SiQDs covered with thin layers of the acid-related coating (Fig. 1b and S2a). Detail structural characterization of the SiQDs can be found in our recent work.^20,22^ The Textural properties of all SiQDs powder can be seen at Table S1. We also observed good dispersion of QDs during the treatment as demonstrated by TEM image of QDs within PhA prior to the removal of the excess acid (Fig. 1b, right inset). The only difference in morphology between the treated samples occurred when using both acids, which exhibited larger aggregates of organic material (Fig. S2a). This is firstly due to the character of PhA, which is a well-known cross-linking agent widely used for the formation of hydrogel-based materials. When it is combined with PAA, it interacts through hydrogen bonding and electrostatic interactions forming cross-linked structures. Secondly, the combined amount of both acids is twice as high as in the case of surface treatment with only one of the acids due to the requirement of preserving the concentrations. Thus, the increased amount of both acids together with their cross-linking abilities results in the formation of bigger aggregates.^27,28^ It is worth mentioning that at a pH ∼3 (corresponding to the pH of the final suspension PAA + PhA + SiQDs), the interaction between PAA and PhA is dominated almost entirely by hydrogen bonding, as both molecules are highly protonated and carry very few ionic charges. At this acidity, the carboxylic acid groups (–COOH) of PAA are fully protonated, making the polymer largely neutral, while most of the phosphate groups (–PO_4_H_2_) in PhA are also protonated, reducing its overall negative charge. Because of this, electrostatic attraction is minimal, and instead, dense hydrogen-bond networks can form between the hydroxyl and acidic protons of both molecules.

These interactions can lead to physical association or weak gel-like structures, but they remain non-covalent and reversible. The high proton concentration also tends to suppress ionization and solubility, potentially causing phase separation or precipitation if concentrations are high. In summary, at pH ≈ 3, PAA and PhA primarily interact through strong hydrogen bonding with limited ionic contribution, forming weak, reversible complexes that are sensitive to even small increases in pH.

The time-integrated photoluminescence (PL) can serve as a quick and non-invasive tool to monitor the changes occurring on the surface of SiQDs, because their PL is highly sensitive to surface chemistry and the distribution of charges. While the untreated H-SiQDs exhibited low-intensity infrared (IR) emission (Fig. 1c), both air-induced oxidation (pO-SiQDs) as well as acid treatment led to a blue shift in the PL spectrum and an increase in PL emission efficiency. This is consistent with the typical behavior of SiQDs, where oxidation leads to the passivation of surface defects, improving the radiative performance and shrinking of the core resulting in the blue-shift of the observed PL. Both PAA-treated H-SiQDs (with and without PhA) samples showed PL characteristics similar to untreated pO-SiQDs, but in the PhA terminated sample the blueshift was not so significant. These results indicate effective surface oxidation of the H-SiQDs in the presence of both acids and suggest that PhA-treated H-SiQDs may retain a degree of the original hydrogen termination even after acid exposure. Samples in the pO-SiQDs series exhibited PL behavior comparable to that of the original material, see Fig. S2b, indicating further surface oxidation during interaction with the acid.

The analysis of the surface chemistry of H-SiQDs series was obtained using Fourier-transform infrared spectroscopy (FTIR). FTIR data within important spectral regions are shown in Fig. 1d and e, full FTIR spectra within the range of 400–4000 cm^−1^ are presented in Fig. S3a. Four main spectral regions can be identified (Fig. 1d) in the untreated H-SiQDs sample: (i) the first region (580–730 cm^−1^) contains two maxima at 630 and 665 cm^−1^, commonly assigned to the out-of-plane bending modes of *Si–H and/or *Si–H_2_, respectively^20,29^ (the asterisk denotes an Si atom bonded to other Si atoms within the QD structure). (ii) The second region (780–980 cm^−1^) is associated with hydrogen- and oxygen-related surface terminations of Si (Fig. 1d inset).^30–32^ A signal near 800 cm^−1^ can be assigned to stretching vibrations *ν*(*Si–O–*Si), corresponding to bridging or non-bridging oxygen bonds (NBOs).^31^ A doublet at 853 and 904 cm^−1^ is typically attributed to one or both bending *δ*(*Si–H_2/3_) vibrational modes.^20,32^ However, as the 853 cm^−1^ peak appears exclusively in SiQDs synthesized *via* non-thermal plasma, it's origin can be more plausibly attributed to structures formed through stepwise Si polymerization, which is characteristic of non-thermal plasma synthesis. The peak at around 840 cm^−1^ is likely connected to surface oxidation after which it becomes more prominent.^20^ The peak at 980 cm^−1^ is usually interpreted as the symmetric stretching mode *ν*_s_(*Si–OH).^33^ A more comprehensive analysis of the origin of the FTIR peaks in this spectral region is available in our previous studies.^20^ (iii) The third spectral region, 990–1200 cm^−1^, corresponds to asymmetric stretching vibrations of *Si–O–*Si made up by two in-phase modes (longitudinal and transverse optical modes, LO and TO) and possibly an out-of-phase mode.^33^ Importantly, the *Si–O vibration is relatively strong in this spectral region due to a significant change in the dipole moment. Thus, the presence of pronounced *Si–O-related peaks does not necessarily reflect a high level of oxidation, as even small amounts of *Si–O can produce strong infrared signals. (iv) The final region, 2000–2200 cm^−1^, is related to the symmetric stretching modes of *ν*_s_(*Si–H_*x*_) surface bonds and brings information about the composition of surface hydrides.^20,34^

The untreated H-SiQDs sample exhibited only *Si–H-related surface vibration and a small degree of oxidation, some of which might have occurred simply during the FTIR measurement. The treatment of H-SiQDs by PAA led to a decrease in Si–H_*x*_ bonds concentration (region i, ii, iv), which were transformed into Si oxides (signal at 990–1200 cm^−1^). The presence of PAA in the sample was confirmed by characteristic PAA peaks (Fig. 1e), specifically around 1350 cm^−1^, 1450 cm^−1^, and 1700 cm^−1^, which correspond to *ν*(C–O), *δ*(CH_2_), and *ν*_s_(C=O) vibrations, respectively. Additionally, peaks at 840, 870, and 1620 cm^−1^ which are not observed in untreated H-SiQDs or pure PAA, appear. The first peak (840 cm^−1^) is most likely related to the surface oxidation of the SiQDs, while the last peak (1620 cm^−1^) can be attributed to *ν*_as_(C=C) vibrational modes, potentially resulting from the non-ideal course of the polymerization of PAA. The peak at 870 cm^−1^ can be assigned to *ν*_s_(*Si–O–C). This assignment can be further supported by a clear modulation of the peak located at 1100 cm^−1^,^35^ which his dominated by *ν*_as_(*Si–O–*Si), but includes contributions from *Si–O–C bonding. The presence of *Si–O–C indicates the formation of a covalent bond between PAA and the SiQDs most probably through the dehydrogenation reaction of PA carboxyl groups with the surface *Si–H_*x*_.

Covalent *Si–O–C bonding is somewhat unexpected. Although the formation of this bond on SiQDs surfaces has previously been observed in radical-initiated reactions under UV irradiation or *via* hydrosilylation, such processes were conducted under highly controlled conditions that suppress or neglect competing surface oxidation.,^36,37^ as noted in the Experimental section, the PAA treatment results in a slurry with a pH of approximately 5, which is above the p*K*_a_ of PAA (∼4.5). According to the speciation diagram, under these conditions, roughly 75% of the PAA carboxylic groups are deprotonated, favoring ionic interactions with the SiQDs surface (for calculation see SI).^15^ Typically, ionic bonding of deprotonated carboxylates to the surface is accompanied by a shift in the *ν*_s_(C=O) group near 1700 cm^−1^. In our samples, we indeed observed a ∼10 cm^−1^ shift in this band, which supports the presence of such interactions. However, our FTIR spectra also showed the *ν*_as_ (*Si–O–C) vibration, indicating the formation of covalent bonds between PAA and the H-SiQDs surface. Thus, both ionic and covalent *Si–O–C bonding are present in PAA-H-SiQDs. We attribute the occurrence of covalent bonding to the high concentration of reactive Si–H_*x*_ surface groups in our H-SiQDs, which, unlike in conventional Li-ion anode preparations where oxidation is more prevalent, can directly react with the residual 25% of protonated carboxylic acid groups in PAA. This reactivity in turn allowed covalent ester-like linkages (Si–O–C) to form. No other chemical species were detected in the FTIR spectra, further supporting the selectivity of the PAA-H-SiQDs surface chemistry.

The treatment of H-SiQDs with PhA also led to a decrease in the Si–H_*x*_ signal and an increase in the signal associated with *ν*_as_(*Si–O–*Si), while the *δ*(Si–H) mode was partially preserved. This observation agrees with PL measurements documenting a partial preservation of surface hydrogenation of SiQDs. Demonstrating the presence of PhA in the sample after the removal of excess acid is challenging, as the strong *ν*_as_(P–O–P) vibration is masked by the dominant *ν*_as_(*Si–O–*Si) band. However, weak signals around 1650 cm^−1^, associated with the vibration of P=O(OH) can still be detected. The spectrum of PhA-H-SiQDs was dominated by a distinct signal at 823 cm^−1^, which was not observed in either SiQDs or pure PhA. While this band appeared within the range usually attributed to *ν*(*Si–O–*Si), the very similar atomic masses of Si and phosphorus suggest that the vibration may originate from an *Si–O–P bond. If it was the case, the *ν*(*Si–O–P) mode would be located between the bands of *ν*_as_(*Si–O–*Si) and *ν*_as_(P–O–P) and its position would be shifted by about 20 cm^−1^ below the *Si–O–*Si vibration.^38^ Both of these conditions are fulfilled and, moreover, an analogical assignment of *a* ≈825 cm^−1^ FTIR peak with an *Si–O–P vibration was introduced in the study of the synthesis of silica–phytic acid hybrid gels.^39^ Even though an unambiguous attribution of this signal to an *Si–O–P bond is further complicated by its limited stability in aqueous environments due to potential reverse hydrolysis.^40^ The stability can be significantly enhanced by the local bonding environment, namely by the highly distorted surface of Si nanoparticles.^20,40^ Similarly to the case of *Si–O–C, even though the corresponding *ν*_as_(*Si–O–P) was masked by the dominant *ν*_as_(*Si–O–*Si) signal in our spectra,^35^ a modulation of the band was still observed, supporting our assignment. Therefore, the treatment of H-SiQDs with PhA also led to the covalent attachment of the PhA molecules onto the QDs' surface, resulting from a chemical reaction analogical to the case of *Si–O–C, which was accompanied by partial oxidation.

The FTIR analysis of H-SiQDs treated with a combination of acids then confirmed the successful surface attachment of both acids, as evidenced by the presence of both *Si–O–C and *Si–O–P bonds. However, the intensities of the relevant peaks were lower, especially of the one related to the *Si–O–P bonds, suggesting lower surface coverage. The lower surface coverage was caused by the agglomeration promoted by the presence of both acids, which in turn diminished the effective surface area available for binding interactions. Moreover, aggregation limited the efficient binding of organic ligands, thereby facilitating more extensive surface oxidation.

As expected, the untreated pO-SiQDs exhibited a lower surface coverage by Si–H groups and a higher degree of oxidation compared to the untreated H-SiQDs, demonstrated by increase in the *ν*_as_(*Si–O–*Si) to *Si–H ratio in region (iv) from 1.17 to 2.5 (Fig. 1f). For full spectra within interval of 400–4000 cm^−1^ see Fig. S3b. The surface chemistries of the treated pO-SiQDs samples displayed similar composition as those observed in the H-SiQDs series, particularly a further increase of the signal related to the *Si–O bonds, a significant decrease in the Si–H_*x*_ bonds with some partial preservation of hydrogen termination when the PhA acid was used. One of the few differences between the two series was a reduced intensity of the *Si–O–P and *Si–O–C bonds in the case of the pre-oxidized pO-SiQDs. This can be attributed to the formation of these linkages predominantly *via* Si–H groups, which are less abundant in pO-SiQDs. This observation underscores the necessity of surface hydrogenation in order to achieve stable covalent attachment of the functionalization agents.

### 3.2 XPS analysis

To complement the analysis of the chemical and structural effects of acid treatment on SiQDs, X-ray photoelectron spectroscopy (XPS) was employed. While FTIR provides general information about the degree of oxidation and the nature of surface termination, XPS enables a quantitative analysis. The XPS spectra are dominated by the peaks related to oxygen (O, 533 eV) and Si (100–105 eV) (see representative spectra in Fig. S4). In addition, typical carbon (C) contamination (285 eV) was observed, along with minor nitrogen (N, 402 eV) and fluorine (F, 690 eV) impurities of unknown origin. Notably, trace amounts of phosphorus (P, ≈ 135 eV) were also detected. These spectra were used to evaluate the elemental compositions (averaged over three measurements) and to calculate the ratio of Si^0^ to Si^4+^ oxidation states quantifying the varying degrees of oxidation (Table 1). For the deconvolution of the detailed lines, components consisting of a combination of Gaussian and Lorentz curves were used with the FWHM range set analogical to the measurements of Ag3d_5/2_ lines at the same machine setting. Based on the statistical analysis of repeated measurements, the relative error of the obtained values was estimated to be around 5% of the reported values.

**Table 1:** XPS-based elemental analysis using atomic %. The ratio of Si atoms in non-oxidized (Si^0+^) and fully oxidized (Si^4+^, corresponding to SiO_2_) forms as well as the value of the degree of oxidation *D*_o_ for all samples are also presented. The overall atomic % does not give 100% while the contamination of mainly nitrogen atoms is not presented

Sample	Si (%)	O(%)	P(%)	C(%)	Si^0+^ : Si^4+^	*D*_o_ (%)
H-SiQDs	18.5	21.3	—	59.0	—	8
PAA-H-SiQDs	29.3	53.0	—	17.0	0.40	87
PhA-H-SiQDs	27.7	56.7	1.3	10.0	0.27	96
PAA + PhA-H-SiQDs	38.8	39.6	0.3	20.5	0.59	77
pO-SiQDs	44.8	28.8	—	25.6	2.45	35
PAA-pO-SiQDs	28.6	59.5	—	11.3	0.22	100
PhA-pO-SiQDs	35.5	59.1	0.1	5.8	0.41	87
PAA + PhA-pO-SiQDs	31.0	51.4	0.2	17.3	0.58	77

First, we intended to use the concentrations of Si and O atoms to calculate the oxidation level of our samples. However, the measured O concentration can be affected by surface contamination from carbon-based species that may also contain O. Therefore, we chose to deduce the oxidation level from the relative concentrations of Si^0^ and Si^4+^ species. This ratio is independent of surface contamination and reflects the proportion of Si atoms with all valence electrons shared with other Si atoms (Si^0+^) *versus* those fully oxidized, as in SiO_2_ (Si^4+^). In our samples, this ratio ranges from 2.45 for pO-SiQDs to about 0.22 for PAA-pO-SiQDs (Table 1). The H-SiQDs did not show presence of any Si^4+^. To quantify the oxidation level more precisely, we introduce the parameter “degree of oxidation” (*D*_o_), defined as follows:1
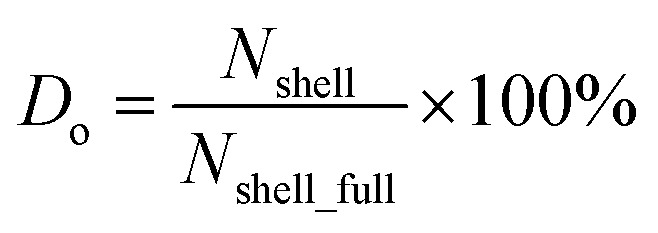


Representing the ratio of the already oxidized Si atoms (*N*_shell_) to the total number of Si atoms that would be present in a fully developed SiO_2_ shell (*N*_shell_full_). The value of parameters *N*_shell_ and *N*_shell_full_ can be estimated based on the known SiQDs atomic structure, the Si^0+^ : Si^4+^ ratio, and the ratio of Si^0+^ : Si^4+^ for a fully developed SiO_2_ shell (0.22 in our case; for details of the *D*_o_ calculation, see SI). Using this method, we determined that pO-SiQDs have a *D*_o_ of about 35%, and the remaining acid-treated samples show a value from 77% to 100% (Table 1). This trend is consistent with FTIR results. Based on these results, we were also able to estimate that a fully developed SiO_2_ shell have a thickness of approximately 1.15 nm, covering a non-oxidized Si core with a diameter of about 3.2 nm as detected by XPS. Notably, the Si^0+^ : Si^4+^ ratio was not evaluated for the untreated H-SiQDs sample, as it did not exhibit a signal typical of the Si^4+^ species. Instead, only the presence of suboxides was detected, which is characteristic of the earliest stages of SiQDs surface oxidation. To enable a comparison of the oxidation degree of this sample with the others, the *D*_o_ of value about 8% was calculated based on the O-to-Si atomic ratio (see SI). However, this number clearly represents only the upper limit of the stage of oxidation and is related to suboxides, rather than the fully oxidized state in the remaining samples.

While the surfaces of the non-treated samples were found to be significantly contaminated by C-based species, the validity of the conclusions related to the *D*_o_ calculation was supported by elemental analysis of the PAA-H-SiQDs sample, which is expected to exhibit minimal surface contamination due to the polar nature of PAA. Assuming that all the detected C in this sample originates from PAA, based on chemical composition of PAA (3× C and 2× O for monomer) we can estimate the ratio of Si to O atoms associated solely with surface Si oxidation (*i.e.* the PAA-related O was subtracted) to be 0.705 (see elemental analysis of Table 1). The calculation assumes a homogeneous structure of the sample, and no correction based on the differences in the escape depths of the C 1s and O 1s photoelectrons, or on the structural characteristics of the polymer layer, was applied. This value is very close to the expected value of 0.697 which would be related to SiQDs sample with a *D*_o_ of 87%, supporting both the reliability of the *D*_o_ parameter and the low C-based contamination of the sample (for calculation of *D*_o_ from ratio of Si : O, see SI and SEQ 4). Applying the same evaluation method to PhA-H-SiQDs resulted in a slightly higher Si : O ratio based on elemental analysis compared to the value from the *D*_o_ parameter calculation, indicating that the detected C atoms cannot be attributed solely to PhA and that additional C-based contamination is present in this case. Since the ratio of PhA to PAA is unknown in samples treated with both acids, accurate analysis of C-based contamination of samples treated by both acids is not possible. A similar overall conclusion regarding the samples' oxidation levels and presence of C-based surface contamination applies to the second series of samples as well (Table 1).

Aside from Si, O, and C, we also detected a small but important amount of P in the PhA-treated samples (Table 1). Considering that a single SiQDs is made up by approximately 4400 Si atoms (based on their size and faceting)^41^ and each PhA molecule contains six P atoms, the P concentration of 1.3% observed in the PhA-H-SiQDs sample corresponds to roughly 35 PhA molecules per SiQDs. Based on our recent findings, H-SiQDs used in our study exhibit surface-dominant faceting along the {111} crystallographic planes with ideally 750 Si–H bonds on each typical QD (density of surface states on SiQDs of {111} is about 7.80 × 10^14^ cm^2^).^20,39,41^ Considering this, along with the high surface curvature of the SiQDs and the molecular size of PhA, even such a low concentration of P corresponds to a relatively high surface coverage. In other samples, the PhA concentration is much lower, corresponding to only a few PhA molecules per SiQD (roughly two molecules per 0.1% P detected). Even though this value is almost at the detection limit of the instrument implying a high degree of uncertainty, these results are consistent with the FTIR analysis, confirming surface-bonded PhA only in the case of PhA-H-SiQDs.

### 3.3 Sensitivity of the SiQDs to pH

**Fig. 2:**
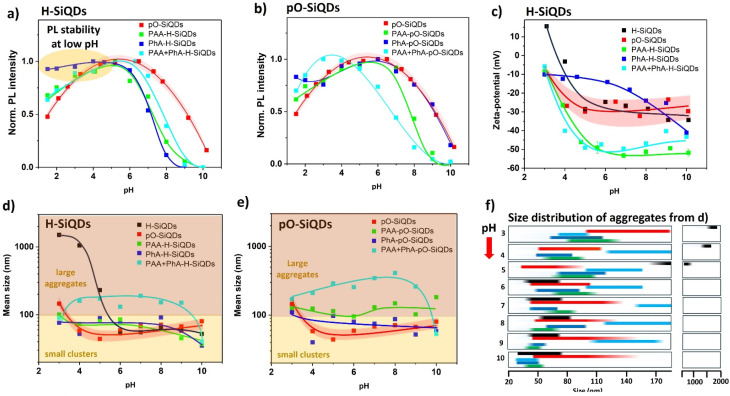
(a and b) Normalized PL dependence on pH of the H-SiQDs and pO-SiQDs series dispersed in water, respectively. The pO-SiQDs sample was used as a reference in both cases because of low PL intensity in the untreated H-SiQDs. (c) Zeta-potential dependence on pH of the H-SiQDs series and untreated pO-SiQDs dispersed in water. (d and e) The pH dependence of the mean size of samples from (b and c) dispersed in water as determined by DLS. (f) The detailed size distribution of the samples from panel (d). The position and width of each bar reflect the corresponding size distribution curve obtained by DLS measurements. Bar colors match those used in panel (d) for consistency. The curves in panels (a–e) serve as guides-to-the-eye. The standard deviation of the zeta-potential maxima was approximately 20% of the measured value, 10–15% for the DLS results, and about 5% for the PL intensity measurements. For the sake of clarity, the measurement uncertainties are presented only for one sample (pO-SiQDs) in each panel using its confidence band (66%).

A property inherently linked to the fabrication of battery anodes is pH stability. Therefore, we investigacted the influence of pH on PL properties, surface charge (using zeta-potential measurements), and size distribution in dispersions (*via* DLS) of the samples studied (Fig. 2). Due to the occurrence of irreversible surface alterations in the samples at pH levels exceeding 7, the measurements were realized by dividing each sample into two portions for subsequent analysis. The first portion was examined under progressively decreasing pH conditions, while the second one was tested under increasing pH conditions starting from the initial pH value, which varied 5–6.5 depending on the acid used for sample preparation.

The pH dependence of normalized PL intensity maxima of the samples from the H-SiQDs series is shown in Fig. 2a. The untreated pO-SiQDs are used as a reference here, since the untreated H-SiQDs exhibited a far too low PL response. The pH dependences of all samples including pO-SiQDs showed a comparable behavior: the highest PL intensity was observed at around pH 6, with PL quenching occurring both at lower and higher pH levels. The spectral position of PL showed a small blue shift with a sharp change after crossing pH 9 (Fig. S5a). Although the interpretation of PL changes during pH reduction is not straightforward, the quenching of PL in pO-SiQDs at pH levels above 7 can be attributed to rapid, irreversible silicon oxidation. This process leads to the formation of defect states that quench the PL emission and is subsequently followed by the dissolution of the SiO_2_ shell, as described by the following reaction:^42^

2a Si + 2OH^−^ + H_2_O → SiO_2_^2−^ + 2H_2_

2b SiO_2_ + OH^−^ → Si(OH)_6_^2−^

This chain of chemical reactions in turn further exposes vulnerable *Si–Si bonds previously hidden and protected in SiQDs core. The proposed mechanism of the gradual corrosion of the Si core also matches the observed blue shift in the PL spectrum, which is usually linked to a decrease in SiQDs size. As the pH increases, the efficiency of this reaction improves, potentially resulting in the complete degradation of SiQDs.^43^ In comparison with the untreated pO-SiQDs, all the acid-treated H-SiQDs samples showed an even faster decrease of PL intensity with increasing pH. It is thus evident that the acid-based surface termination renders SiQDs even more sensitive to increasing pH levels. Interestingly, when the pH change occurred in the opposite direction, acid termination appeared to maintain the PL intensity. While the non-treated SiQDs decreased their PL efficiency by more than 50% after reaching pH 1.5, PL intensity of H-SiQDs samples treated by PAA (solo or in combination with PhA) still kept about 70% of the initial value and PhA-H-SiQDs exhibited only a negligible drop of PL with decreasing pH of the environment.

In the pO-SiQDs series (Fig. 2b), the overall PL dependence on pH was similar. However, the PhA-pO-SiQDs sample exhibited greater stability at higher pH, with performance comparable to the untreated pO-SiQDs reference, likely due to a reduced number of *Si–H_*x*_ surface bonds susceptible to core-corroding oxidation. At lower pH, its PL preservation was slightly less effective than that of PhA-H-SiQDs, stabilizing at approximately 85% of the original intensity. The pO-SiQDs treated with both acids showed similar pH-dependent PL trends as observed for the same sample based on H-SiQDs, although the PL maximum was shifted toward a lower pH.

Zeta-potential measurements across a pH range of 3 to 10 for the H-SiQDs series are compared to pO-SiQDs in Fig. 2c. Even in this case, all samples from the H-SiQDs series, except the one treated with PhA, exhibited a similar trend. They displayed negative values of surface charge (about −30 mV non-treated SiQDs; −55 mV treated) with rapid neutralization of surface charge occurring after exceeding pH 5. The PhA-H-SiQDs sample exhibited a zeta potential of approximately −10 mV at pH 3, which remained relatively stable up to pH 6. Beyond this point, a progressive increase in negative surface charge was observed, reaching −40 mV at pH 10. Samples from the pO-SiQDs series displayed analogous pH-dependent behavior. However, the PhA-treated variant was even less responsive to pH changes, with a final zeta potential of −20 mV at pH 10 (Fig. S5b).

The mean hydrodynamic diameters (labelled as mean size) obtained from dynamic light scattering (DLS) measurements over the same pH range were consistently larger than the primary size of the as-synthesized SiQDs (∼5.5 nm), indicating the formation of aggregates upon dispersion in water. These aggregates typically consisted of smaller aggregates (<100 nm) and larger assemblies (100 nm −1 µm), which together formed the volumetrically dominant fraction of the dispersed quantum dots and heterostructures. The pH dependence of the mean aggregate size for both untreated SiQDs types was consistent with the zeta potential trends, showing an increase in size from ∼60 nm at high pH to ∼200 nm and ∼1500 nm at low pH for pO-SiQDs and H-SiQDs, respectively (Fig. 2d). In contrast, acid-treated samples exhibited minimal sensitivity to pH variation, maintaining mean sizes of approximately 70 nm for both PAA- and PhA-treated SiQDs, with a slight reduction observed above pH 9. Treatment with both acids, however, resulted in aggregates around 200 nm in size, suggesting significant interparticle interconnection likely mediated by PAA and influenced by the sequence of treatment steps. In the dual-acid protocol, SiQDs were first physically mixed with PAA, followed by dispersion and sonication in water, during which an aqueous solution of PhA was introduced. Because PhA was not present during the initial mixing stage, its subsequent addition may have resulted in weaker direct interaction with the SiQDs surfaces and stronger interactions with the pre-adsorbed PAA, thereby promoting the formation of more interconnected aggregates. Additional insight into the aggregation behavior is provided by the corresponding size distribution profiles (Fig. 2f).

The size distribution of H-SiQDs was relatively narrow (∼30 nm), indicating well-defined collective behavior likely governed by variations in coulombic interactions between the particles. A similarly narrow distribution was observed for H-SiQDs treated with either PhA or PAA. In contrast, pO-SiQDs and PAA + PhA-treated H-SiQDs exhibited broader size distributions, spanning approximately 80 nm, which remained largely insensitive to pH changes, except under extreme conditions. This behavior suggests that aggregation in these systems is not primarily driven by electrostatic interactions but rather by strong oxygen bridging between SiQDs or by intermolecular linkages involving PAA and PhA attached on the SiQDs surfaces. The pO-SiQDs series demonstrated comparable trends in both mean size and size distribution. Notably, these samples displayed markedly broader size distributions and increased aggregate dimensions overall. In both samples containing PAA, aggregates extending up to 500 nm were also observed (Fig. 2e and S6).

## 4. Discussion

**Fig. 3:**
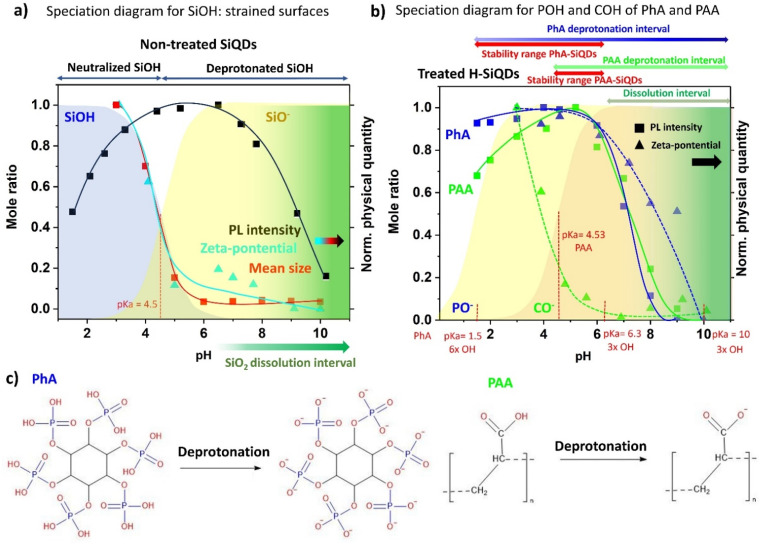
(a) Speciation diagram of SiOH on the surface of non-treated SiQDs. Normalized pH dependence of PL intensity of pO-SiQDs and zeta-potential and mean size of aggregates of H-SiQDs in water are also included. The p*K*_a_ value of SiOH on nanostructured surfaces is indicated. (b) Speciation diagram of POH and COH of PhA and PAA, respectively. Figure also contains normalized dependence of PL intensity and zeta-potential of H-SiQDs terminated by Pha and PAA and dispersed in water. The p*K*_a_ values of PhA and one for PAA are also indicated. (c) Scheme of PhA and PAA in neutralized and fully deprotonated form.

First, since the interpretation is not completely straightforward based on published literature, we will discuss the phenomena occurring in the untreated SiQDs using the speciation diagram, which describes the change of the form of the dissolved species around a p*K*_a_ value with 1 : 1 ratio. A speciation diagram for SiOH groups on the highly strained and curved surfaces of our SiQDs, differing significantly from that of bulk silicon, with the average p*K*_a_ value of 4.5,^44^ is presented in Fig. 3a (for details about the calculation of a speciation diagram based on Henderson–Hasselbalch eqn, see SI) together with the pH dependence of the normalized PL intensity at maximum, zeta-potential and mean size measured in non-treated SiQDs (Fig. 3a). The pH dependence of the zeta-potential and the mean size in untreated H-SiQDs almost perfectly match the neutralization curve, suggesting that the deprotonation and neutralization processes are responsible for the change of surface charge and agglomeration of H-SiQDs. Perhaps more surprisingly, the pH dependence of PL also well correlates with the neutralization curve. At high pH, the PL is clearly quenched by the dissolution of the Si core. At low pH, the neutralization of surface SiOH groups alters the charge distribution around the nanoparticles by enhancing the influence of the surrounding positive charge from solvated H^+^ ions. Due to the covalent nature of the SiQDs core, the electron wave function is highly sensitive to both the electronegativity of surface terminations and the local electric field at the surface.^45^ Thus, we propose that both surface charge neutralization and the external electrostatic environment affect the spatial distribution of electron and hole wave functions, modulating their overlap and ultimately influencing the radiative recombination processes. This observation is particularly interesting for the PL characterization, because it implies that a simple PL measurement can serve as a probe to the charge status of SiQDs. Similar tendencies as those in untreated H-SiQDs were also observed in untreated pO-SiQDs (Fig. 2c–f). The differences in agglomerate sizes at low pH between the two non-treated samples are most likely attributable to the higher density of surface *Si–O bonds on the pO-SiQDs, which limit the Coulombic-driven agglomeration observed in H-SiQDs (Fig. 2f).

A more intricate situation arises when evaluating the pH-dependent properties of acid-treated SiQDs. In addition to the inherent pH effects described previously, acid treatment not only results in the formation of stable *Si–O–C or *Si–O–P bonds and oxidation of the QD surface, but the presence of the acid coating itself needs to be taken into account (Fig. 1d and e). Consequently, several factors may contribute to the observed behavior: (i) deprotonation/neutralization of surface SiOH groups, (ii) the influence of newly formed *Si–O–C(P) bonds, and (iii) the acid–base activity of hydroxyl (–OH) groups present in the attached acid molecules (Fig. 3c). To isolate and better understand the effect of surface termination under varying pH conditions, a speciation diagram was constructed for the POH and COH functional groups in PhA and PAA (Fig. 3b), respectively. The PAA monomer exhibits a single well-defined p*K*_a_ of approximately 4.5, whereas PhA contains twelve hydroxyl groups that fall into three distinct acidity regimes: six strongly acidic OH groups (p*K*_a_ ≈ 1.5), three moderately acidic groups (p*K*_a_ ≈ 6.3), and three weakly acidic groups (p*K*_a_ ≈ 10).^46^ For clarity, Fig. 3b displays only the deprotonation profiles of both acids, along with their respective p*K*_a_ values, and overlays these with normalized PL intensity and zeta potential data for H-SiQDs treated with PhA and PAA.

Focusing first on PAA-H-SiQDs, their zeta potential exhibits a pH-dependent trend that closely follows the deprotonation profile of PAA. However, this behavior nearly overlaps with that of the surface *Si–OH groups (Fig. 3a), making it difficult to disentangle the respective contributions of *Si–OH and PAA-derived COH groups to the overall surface charge. Considering the significant surface coverage of H-SiQDs by PAA, as observed *via* TEM, and the contrasting zeta potential behavior of PhA-treated H-SiQDs (discussed below), it is likely that PAA dominates the interfacial properties in this case. The effect of PAA treatment on H-SiQDs is also evident in the aggregate size data, which show minimal sensitivity to pH across a broad range, with only a slight decrease at high pH. This behavior can be attributed to the relatively unoxidized, hydrophobic surfaces of H-terminated SiQDs, which possess fewer surface charges than their oxidized counterparts. These surfaces facilitate stronger adsorption of PAA chains through hydrophobic and van der Waals interactions in combination with the already described covalent bonding, while electrostatic repulsion remains minimal. At pH values above 7, PAA exists in a fully deprotonated state, assuming an extended coil conformation that provides effective electrostatic repulsion and steric hindrance, thereby stabilizing the dispersion. The resulting repulsive forces between PAA-coated SiQDs suppress aggregation, leading to the formation of small, well-dispersed clusters. The interpretation of PL intensity behavior in PAA-H-SiQDs is notably more complex. Compared to the untreated sample, the PL intensity of the modified H-SiQDs exhibits a more pronounced decline at high pH. Since the onset of this decrease coincides with that observed for non-treated SiQDs, the reduction in PL is most likely associated with enhanced Si oxidation and SiO_2_ dissolution. This effect may arise from structural changes induced by the acid treatment and/or from the influence of deprotonated acid species. The former could lead to the formation of defect-rich surface *Si–O states that are more susceptible to alkaline dissolution. In parallel, given that dissolution proceeds *via* reaction with OH^−^ ions, the presence of deprotonated hydroxyl groups from the acid may facilitate or accelerate this process. Returning to the interpretation of the effect under low pH conditions, the slow PL intensity decline within this pH interval in the case of PAA-treated SiQDs could be attributed to the presence of stable *Si–O–C bonds, which are not affected by pH change. As a result, these bonds help preserve the spatial distribution of electron and hole wave functions during surface neutralization, thereby maintaining higher PL efficiency in the low-pH regime.

In the case of PhA-H-SiQDs, the pH dependence of zeta potential is clearly governed by the speciation behavior of PhA. The very low p*K*_a_ of half of its hydroxyl groups stabilizes the surface charge across a broad pH range, with a gradual decrease observed upon surpassing the second p*K*_a_ value (Fig. 3b). Similarly, the agglomerate size remains nearly constant, with only a slight reduction occurring at pH values above 8 (Fig. 2d). This reduction can be attributed to the complete deprotonation of PhA's phosphate groups, as supported by the corresponding zeta potential data. The resulting increase in surface charge density enhances electrostatic repulsion between particles, suppressing bridging interactions and thereby improving colloidal dispersion of PhA-coated H-SiQDs. The highest PL intensity is again observed in the pH range of 4–6, followed by a sharp decline above pH 6, likely due to increased SiO_2_ dissolution, as described in the case of PAA-H-SiQDs. Interestingly, PL intensity remains almost entirely preserved even as pH is lowered to 1.5. This behavior cannot be fully explained by the presence of *Si–O–P bonds alone, as their surface concentration is not sufficient (∼35 bonds per QD). More plausible, the adjacent PO^−^ groups, which contribute to the negative charge near the SiQDs surface (Fig. 3c). These negatively charged groups may compensate for the electric field generated by surrounding H^+^ ions, either by slowing the protonation of surface SiOH groups or by mitigating its effect on the distribution of core electron wave functions. In either case, the persistent negative electrostatic environment provided by PhA at very low pH helps maintain the spatial distribution of electron and hole wave functions within the SiQDs core, thereby preserving PL efficiency. The protective role of PhA under acidic conditions is further supported by the partial retention of surface Si–H bonds following acid treatment (Fig. 1d).

The treatment of H-SiQDs with both PAA and PhA did not result in any additional beneficial effects. The PL and zeta potential responses were dominated by the presence of PAA, and the large mean aggregate size was caused by the mutual interactions between PAA and PhA, which can form a complex, cross-linked polyelectrolyte network through multivalent ionic interactions and hydrogen bonding. Such a network likely captures SiQDs within interconnected domains, promoting the formation of larger agglomerates. Furthermore, the overall acid concentration in this dual treatment was twice that of the individual treatments, which likely enhanced inter-particle bridging and further contributed to the observed increase in aggregate size.

For the pO-SiQDs series, the zeta potential exhibited a pH-dependent trend comparable to that of the H-SiQDs series. However, these samples showed larger mean aggregate sizes and broader size distributions (Fig. S5) due to a lower number of bonded acid molecules and the inherent tendency of peroxidized SiQDs to form clusters *via* *Si–O–*Si interparticle linkages. In the case of PAA–pO–SiQDs, the PL response as a function of pH was not significantly altered using pre-oxidized QDs. This is likely because the small size of PA enables good PAA surface coverage and thus sufficient surface bonds are present even after the decrease of the potential *Si–H_*x*_ sites on SiQDs surface. Different situation was observed in the case of PhA–pO–SiQDs. These samples exhibited PL characteristics similar to those of untreated pO–SiQDs at high pH, with only limited preservation of PL at low pH (Fig. 2). This behavior is attributed to the reduced availability of reactive Si–H_*x*_ sites required for forming covalent bonds between PhA and the SiQDs surface. As a result, even after PhA treatment, the optical response at high pH remains largely governed by the intrinsic properties of pO–SiQDs, and only partial PL preservation is observed under acidic conditions. As with the H-SiQDs series, the dual acid treatment did not yield additional improvements. The observed pH-dependent behavior remained dominated by PAA, aside from an increase in aggregate size due to the combined effect of both acids.

Our green approach delivers benefits at every stage of H-SiQDs preparation, termination, and application (for summary see Table 2). First, during synthesis, H-SiQDs are produced by non-thermal plasma, which is currently the only technique not requiring the use of hydrofluoric acid (HF) or other hazardous reagents.^1,20^ Even synthesis procedures described as “green,” such as ball milling or synthesis from rice husks, still require HF either during the synthesis or in the post-treatment to form an H-terminated surface.^47,48^ Our non-thermal plasma-based approach thus removes one of the most hazardous chemicals from the process. Second, in surface functionalization, benign, water-soluble acids are used as ligands and dispersants. The use of PhA or PAA therefore bypasses the ecological drawbacks of common surface termination methods. These include hydrosilylation, which often requires hazardous and/or expensive chemicals such as chlorosilanes and pyrophoric reagents, involve solvent-intensive procedures, and frequently demand considerable time and energy,^1,49,50^ as well as other techniques that can employ highly reactive organolithium compounds.^51^ Third, in terms of stability, the PAA and PhA functionalization provides good colloidal and optical stability even in harsh acidic environments. This allows the particles to retain their photoluminescence without the need for additional hazardous stabilizers (*e.g.* allylamine) or encapsulation methods, which are common in conventional systems.^52–55^ Finally, in Li-ion battery electrode processing, PAA functionalization is compatible with water-based binders such as PAA, carboxymethyl cellulose, and alginate, enabling fabrication without toxic solvents.^56,57^ Conventional methods rely on fluoropolymer binders like polyvinylidene fluoride (PVDF) dissolved in *N*-methylpyrrolidone (NMP), dimethylformamide (DMF), or dimethylacetamide (DMAc), which emit volatile organic compounds (VOCs), require high-temperature drying, and introduce persistent PFAS-type materials that hinder recycling.^56,58,59^ In contrast, PAA-functionalized SiQDs are dispersible in water, eliminating fluoropolymers and hazardous solvents. This approach reduces VOC emissions, lowers energy demand, supports renewable binder systems, and facilitates cleaner recovery of active materials. Beyond the immediate ecological benefits, this compatibility with scalable, water-based electrode fabrication aligns with emerging industrial trends toward solvent-free or aqueous battery manufacturing, thereby strengthening the practical and sustainable potential of the proposed functionalization strategy.

**Table 2:** Ecological benefits of proposed H-SiQDs functionalization approach compared to conventional procedures related to each step of the presented technique

Aspect	Current/Conventional approach	This work (proposed approach)	Benefits
H- terminated SiQDs	Requires HF etching during synthesis or within after-processing	Non-thermal plasma synthesis generates H-SiQDs without HF or other toxic liquids	Avoids highly hazardous HF; safer for workers and environment
Surface functionalization	Complex and/or time/energy procedures often demand toxic/pyrophoric reagents (chlorosilanes, organolithium, *etc.*) in organic solvents	Uses benign, water-soluble acids (PAA, PhA) as ligands in aqueous medium	Eliminates hazardous reagents and organic solvents; renewable or low-toxicity agents
pH & water stability	Poor stability. Needs encapsulation or complex termination procedure using toxic reagents (*e.g.*, allylamine)	PAA/PhA functionalization gives colloidal and PL stability even at low pH	Maintains function in highly acidic water without toxic stabilizers
Li-ion battery/Electrode processing	Commonly relies on fluoropolymer binders (*e.g.*, PVDF, PTFE) dissolved in toxic organic solvents (NMP, DMF, DMAc, THF); VOC emissions during drying; energy-intensive removal of solvents (>120 °C); stabilizers often require toxic amines/surfactants	PAA-functionalized SiQDs enable water-based binder processing	Eliminate fluoropolymers and toxic organic solvents; reduces VOC emissions; simplifies recycling; enables low-energy drying
Scalability and process simplicity	Often requires inert atmosphere, complex purification; limited scalability	Aqueous, air-compatible process with simple work-up	Directly scalable; cost-effective; suitable for industrial adoption

Each of these advances is individually important in reducing the environmental footprint of the proposed technology. Although non-thermal plasma synthesis of H-SiQDs is not a new method, and PAA and PhA have previously been used as SiQDs dispersion agents, the true significance lies in the synergy of all steps. The innovative approach we propose combines safe synthesis, non-toxic functionalization, and acid-resistant stability into a single workflow, offering a green and comprehensive route for creating complex QD-based materials for Li-ion battery applications, as well as QD hybrids with potential applications in bioimaging, medicine, and nanosensing.

## 5 Conclusions

This study demonstrates that the environmentally benign acids PAA and PhA act as effective surface passivating agents for SiQDs in aqueous environments. Traditionally used for nanoparticle dispersion or as cross-linking agents in Li-ion battery technologies, these acids also form covalent or mixed covalent–ionic bonds with SiQDs surfaces, functioning as genuine surface functionalizers. Both react with surface Si–H groups to generate stable Si–O–C or Si–O–P linkages, substantially influencing the particles' chemical stability, aggregation, and photoluminescence. The modification markedly enhances stability under acidic conditions by shielding the SiQDs surface from proton attack, though it does not prevent high-pH-induced dissolution. Steady-state PL spectroscopy, supported by FTIR, XPS, DLS, and zeta-potential analyses, revealed that the protection arises from electrostatic interactions between deprotonated acid groups and ambient ions, which suppress PL quenching. The effect was most pronounced for PhA-treated, hydrogen-terminated SiQDs, maintaining full PL intensity even at pH 1.5, where untreated particles lost ∼50%. The improvement results from PhA's limited dissociation and the high reactivity of hydrogenated Si surfaces, enabling efficient binding without atmospheric control. For partially oxidized SiQDs, the benefits were reduced, highlighting the importance of maintaining hydrogen termination for optimal passivation. Using both acids simultaneously, a common approach in SiQD/polymer hybrid fabrication, yielded less favorable stability and optical performance.

These findings challenge the view that PAA and PhA serve only auxiliary roles and establish them as active agents capable of modulating both dispersion and optoelectronic behavior. Owing to their low cost, ready availability, and non-toxic, water-based nature, this work introduces a simple and sustainable strategy for improving the stability and functionality of SiQDs without relying on hazardous reagents or complex processing. Beyond surface stabilization, the demonstrated acid-based functionalization provides chemically active anchoring sites that facilitate the integration of SiQDs into hybrid materials. The carboxylic and phosphonic acid groups can form covalent or coordinative linkages with polymers, metal oxides, or biomolecules, enabling the construction of multifunctional SiQD-based composites. This versatility highlights the potential of the presented approach as a general platform for creating hybrid nanostructures with tailored optical, electronic, or sensing properties. The approach aligns with the principles of green chemistry and offers a scalable pathway towards environmentally responsible, water-compatible silicon nanomaterials for energy-storage, optoelectronic, and hybrid applications.

## Author contributions

P. G. and F. H. proposed the idea of studying the influence of PAA and PhA treatment on SiQDs. P. G. carried out the DLS, zeta potential, and PL measurements, as well as part of the FTIR measurements, proposed the presented interpretation, and drafted the manuscript. G. Č. performed the acid termination of SiQDs, completed the remaining FTIR measurements, and contributed to the interpretation and manuscript drafting. F. M. synthesized the SiQDs used in the study and contributed to the manuscript preparation. F. H. supervised the research at UCT Prague, contributed to the interpretation of the data and drafting of the manuscript. F. L. performed the XPS measurements, except for the non-treated samples, which were measured by P. S., who also evaluated the XPS data. K. K. contributed to the interpretation, participated in the drafting process, and supervised the research. All authors approved the final version of the manuscript.

## Conflicts of interest

There are no conflicts to declare.

## Supplementary Material

RA-016-D5RA08090H-s001

## Data Availability

The dataset is available at Zenodo repository. The DOI for the repository is https://doi.org/10.5281/zenodo.17099878 or is available using following link: https://doi.org/10.5281/zenodo.17099879. Supplementary information (SI) is available. See DOI: https://doi.org/10.1039/d5ra08090h.
