# Correction: Green and scalable surface functionalization of silicon quantum dots using water-soluble organic acids for sustainable hybrid materials

**DOI:** 10.1039/d6ra90010k

**Published:** 2026-02-11

**Authors:** Pavel Galář, Gabriela Čandová, Filip Matějka, Fatima Hassouna, Abdelghani Laachachi, Petr Sajdl, Kateřina Kůsová

**Affiliations:** a Institute of Physics of the CAS, v.v.i. Cukrovarnická 10 162 00 Prague 6 Czechia galar@fzu.czac; b Faculty of Chemical Engineering, University of Chemistry and Technology, Prague Technická 5 166 28 Prague 6 Czechia; c Luxembourg Institute of Science and Technology (LIST) Maison de l’innovation 5, Avenue des Hauts-Fourneaux Esch-sur-Alzette Luxembourg; d Faculty of Environmental Technology, University of Chemistry and Technology Technická 5 166 28 Praha 6 Czechia

## Abstract

Correction for ‘Green and scalable surface functionalization of silicon quantum dots using water-soluble organic acids for sustainable hybrid materials’ by Pavel Galář *et al.*, *RSC Adv.*, 2026, **16**, 2923–2936, https://doi.org/10.1039/D5RA08090H.

The authors regret that in the author list of the published article, the author names were incorrectly presented, with surnames incorrectly shown before first names. The corrected form of all author names are as shown here.

The Royal Society of Chemistry apologises for these errors and any consequent inconvenience to authors and readers.

