# Supplementary material for: Green and scalable surface functionalization of silicon quantum dots using water-soluble organic acids for sustainable hybrid materials
Source: RSC Adv. 2026 Jan 14;16(3):2923–36. doi: 10.1039/d5ra08090h (PMC12801230; doi:10.1039/d5ra08090h)
Supplement: RA-016-D5RA08090H-s001 [file RA-016-D5RA08090H-s001.pdf]

Supplementary Information (SI) for RSC Advances.
This journal is © The Royal Society of Chemistry 2026

Supplementary information

Calculation of parameter *degree of oxidation* (D_o)

The parameter degree of oxidation (D_o) is used to quantify the oxidation level of silicon quantum dots (SiQDs). It is based on the ratio of already oxidized silicon atoms to the total number of silicon atoms that would be present in a fully developed SiO_2 shell. To perform this calculation, we need the Si^{0+}/Si^{4+} ratio of the SiQDs, the size of the SiQDs, and a reference sample of fully oxidized SiQDs with a complete SiO_2 shell. For the determination of Do, we first calculate the number of atoms in a quantum dot (N_{QD}) using the following equation:[1]

$$d_{QD} = \sqrt[3]{\frac{6}{\pi} V_{atom} N_{QD}}, \qquad (SEQ\ 1)$$

where d_{QD} is the diameter of the quantum dot, and V_{atom} is the volume assigned to a single silicon atom within the QD. For this calculation, we used $V_{atom} = 0.020023\ nm^3$.[1] This equation assumes a spherical shape of the QDs, which is generally valid for plasma-synthesized SiQDs. Using the average diameter of our hydrogen-terminated SiQDs (H-SiQDs), 5.5 nm, we estimate $N_{QD} \approx 4400$ atoms. Given that the theoretical XPS penetration depth is about 10 nm, we assume that all atoms within the QD contribute to the signal. Thus, from the Si^{0+}/Si^{4+} ratio, we can estimate the number of oxidized and non-oxidized silicon atoms. As shown in our measurements (Table 1), the Si^{0+}/Si^{4+} ratio approaches a limiting value of approximately 0.2. As a reference, we use the sample terminated by polyacrylic acid (PAA), which has a ratio of 0.22 and is considered to have a fully developed oxidized shell. By comparing this value with the total number of atoms in a QD, we estimate that in the fully oxidized reference sample, 3606 atoms are located in the shell (N_{shell_full}), while the remaining 794 atoms form the protected core (N_{core_full}). Using **SEQ 1**, we calculate the size of the non-oxidized core to be approximately 3.2 nm, with a shell thickness of about 1.15 nm on both sides, which agrees well with previously published results.[2, 3] The D_o is then calculated as the ratio between the number of oxidized atoms in the sample (N_{shell}) and the number in the fully oxidized reference (N_{shell_full}) as is mentioned in the section 3.2. of the manuscript (Equation 1). Using **Equation 1**, non-oxidized samples yield $D_o = 0\%$, while fully oxidized samples give $D_o = 100\%$. This method is not sensitive to the overall elemental composition of the QDs, which may be influenced by oxygen from sources unrelated to silicon oxidation. It is applicable to measurements where the silicon-based XPS signal can be approximated using the Si^{0+}/Si^{4+} components.

Difficulties arise in the analysis of the H-SiQDs sample, where no Si^{4+} signal was detected only features corresponding to suboxides were observed (**Figure S1a**). To accurately deconvolute the Si 2p signal and distinguish between Si^{0+} and suboxide contributions, we performed XPS measurements using two different charge compensation settings. These settings differentially affect the more conductive elemental silicon (Si^{0+}) and the less conductive silicon suboxides, allowing better spectral separation. This approach revealed a concentration ratio of approximately 74% Si^{0+} to 26% suboxides. To determine the nature of the suboxide species, we examined the concentrations of oxygen and carbon atoms, along with the C 1s XPS spectrum (surface contamination, **Figure S1b**). Based on literature references and the measured oxygen content, the C 1s signal was deconvoluted into distinct components: the peak at 288.6 eV corresponds to carboxyl groups (COO^-), while the peak at 286.3 eV is attributed to carbon

atoms bonded to a single oxygen atom (e.g., –C–OH). By comparing the atomic concentrations of carbon and oxygen and evaluating the relative contributions of each, we find that the majority of oxygen is bonded to carbon. Only a relatively small portion remains available for bonding with silicon, suggesting an approximate stoichiometry of $SiO_{0.5}$. While the 74:26 core:shell ratio of silicon atoms would nominally correspond to a D_o of ~31%, this estimation assumes fully oxidized (Si^{4+}) states. Considering that, on average, the "oxygen-affected" silicon atoms exist predominantly in the form of Si^{1+} (rather than Si^{4+}), we recalculated the oxidation degree by comparing the H-SiQDs to a hypothetical sample containing the same number of oxygen and silicon atoms, but with all oxygen bound in Si^{4+} states. This yields a corrected D_o value of approximately 8% (**Table 1**).

We have also been able to calculate D_o from the ratio of Si:O (R_{Si}) atoms when we expect that oxygen is only related to SiO_2. In that case, the D_o can be calculated as follows:

$$D_o = \frac{N_{QD}}{2 \cdot N_{shell\_full} \cdot R_{Si}} \cdot 100\ \%. \qquad (SEQ\ 2)$$

The ratio between silicon and oxygen atoms in partially oxidized Si quantum dots (SiQDs) can be determined in two ways. One approach is to directly compare the elemental composition obtained from XPS measurements. However, it is important to consider that a portion of the detected oxygen atoms may be associated with surface carbon termination or contamination. However, it is often more challenging to determine how many of the detected oxygen atoms are involved in bonds with carbon atoms.

Alternatively, the ratio can be calculated using the relative amounts of oxidized and non-oxidized silicon atoms, from which the degree of oxidation (Do) parameter is derived. The ratio between silicon and oxygen atoms is given by the following relation:

$$\frac{Si}{O} = \frac{N_{QD}}{2 \cdot N_{shell}} = \frac{N_{QD} \cdot 100\%}{2 \cdot D_0 \cdot N_{shell_{full}}}. \qquad (SEQ\ 3)$$

Here, the denominator $2 \cdot N_{shell}$ represents the total number of oxygen atoms in the oxidized shell. Subsequently, the number of silicon atoms in the oxidized shell is substituted using its expression from **Equation 1**.

Calculation of the speciation diagram

The ratio between the protonated and deprotonated forms of the OH functional group of a substance in an aqueous environment at a specific pH is governed by the Henderson-Hasselbalch equation, which has the form:

$$pH = pK_a - \log\left(\frac{[HA]}{[A^-]}\right), \qquad (SEQ\ 4)$$

where [HA] and [A−] represent the concentrations of the protonated and deprotonated forms of the substance, respectively, and pK_a is the value indicating the pH at which the ratio of these concentrations is equal to 1. The pH dependence of the concentrations of the protonated and deprotonated forms is presented using a so-called speciation diagram. This diagram shows how the mole fractions of [HA] and [A−] change with pH. The mole fractions of the protonated form (f_{HA}) and the deprotonated form (f_{A-}) in the speciation diagram are calculated as follows:

$$f_{HA} = \frac{[HA]}{[HA] + [A^-]} = \frac{1}{1 + 10^{pH - pK_a}}, \qquad (SEQ\ 5a)$$

$$f_{A^-} = \frac{[A^-]}{[HA] + [A^-]} = \frac{10^{pH - pK_a}}{1 + 10^{pH - pK_a}}. \qquad (SEQ\ 5b)$$

If the substance contains multiple OH groups with different stabilities, a separate speciation diagram is calculated for each group.

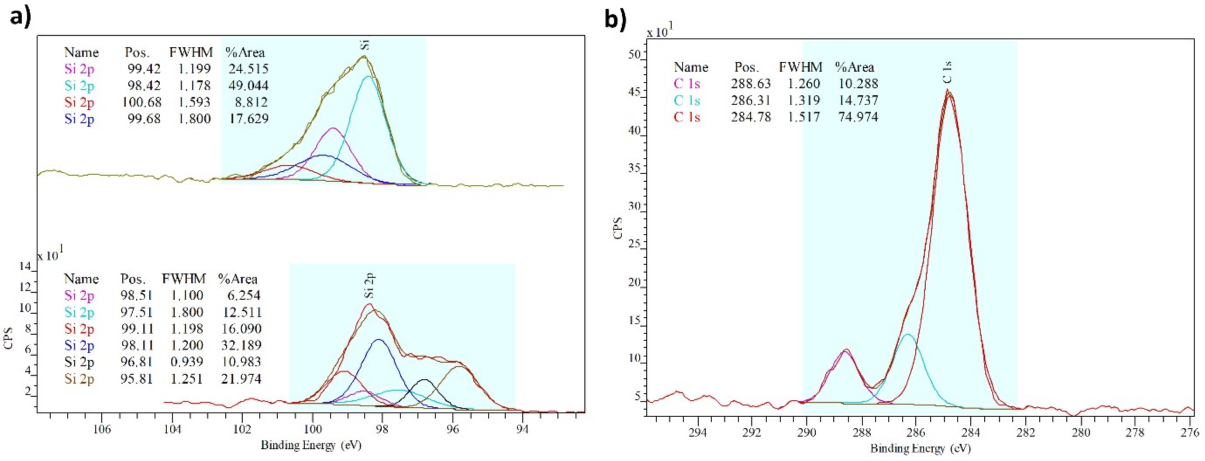

Figure S1. a) XPS measurements of Si 2p band of H-SiQDs sample using two various values of charge compensation. The deconvolution analysis is presented. b) Analysis of C1s band of the sample. The deconvolution analysis is presented.

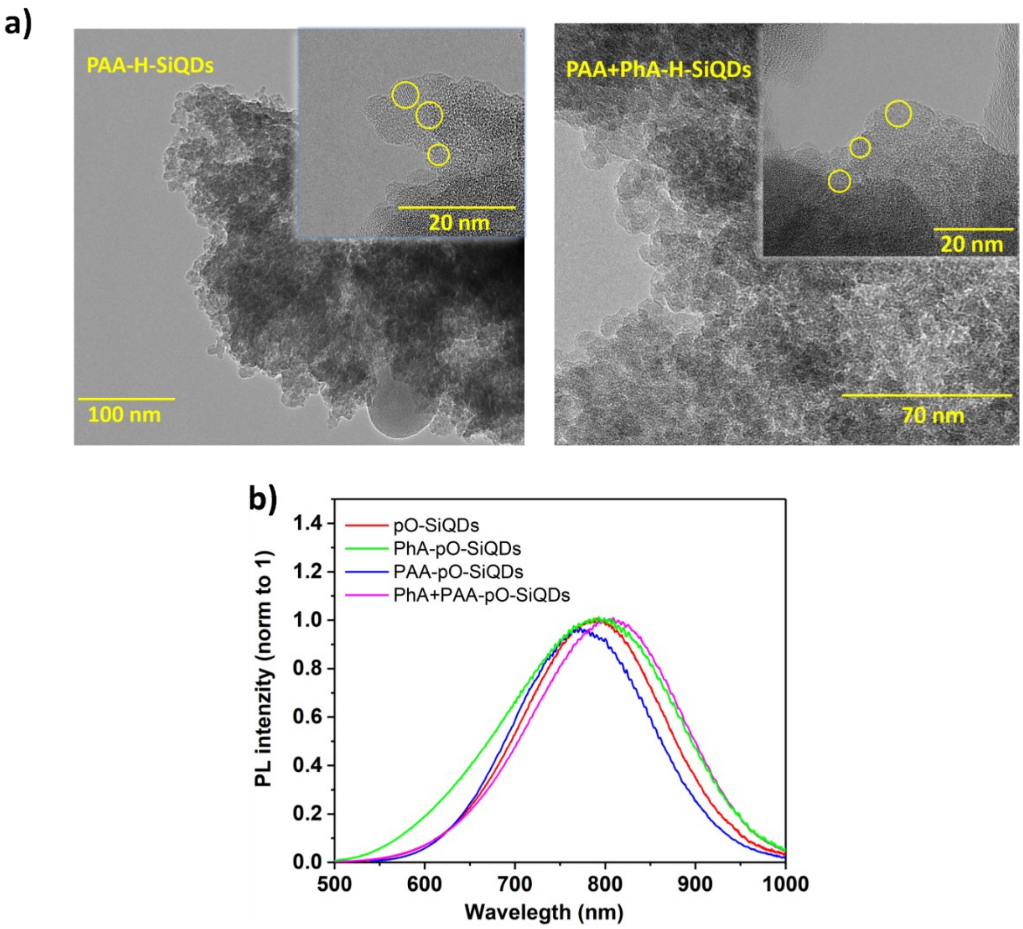

Figure S2. a) TEM images of PAA-H-SiQDs (left) and PAA+PhA-H-SiQDs. Insets display a magnified view of individual SiQDs. b) Normalized PL spectra of samples from the pO-SiQDs series.

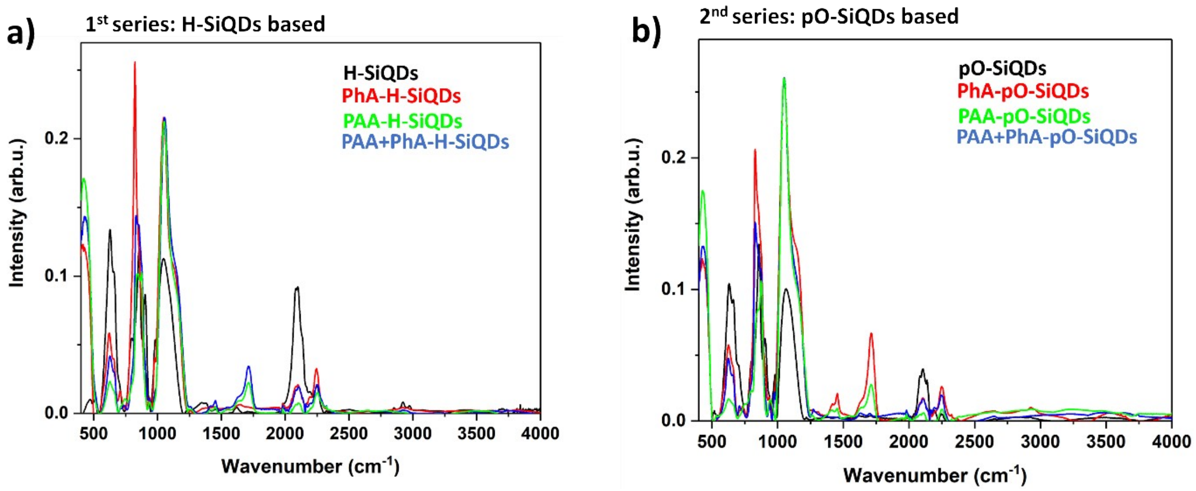

Figure S3. a) FTIR spectra of the H-SiQDs series within the full wavenumber interval ranging 400 – 4000 cm^{-1}. b) FTIR spectra of the pO-SiQDs series within the same interval as presented in a)

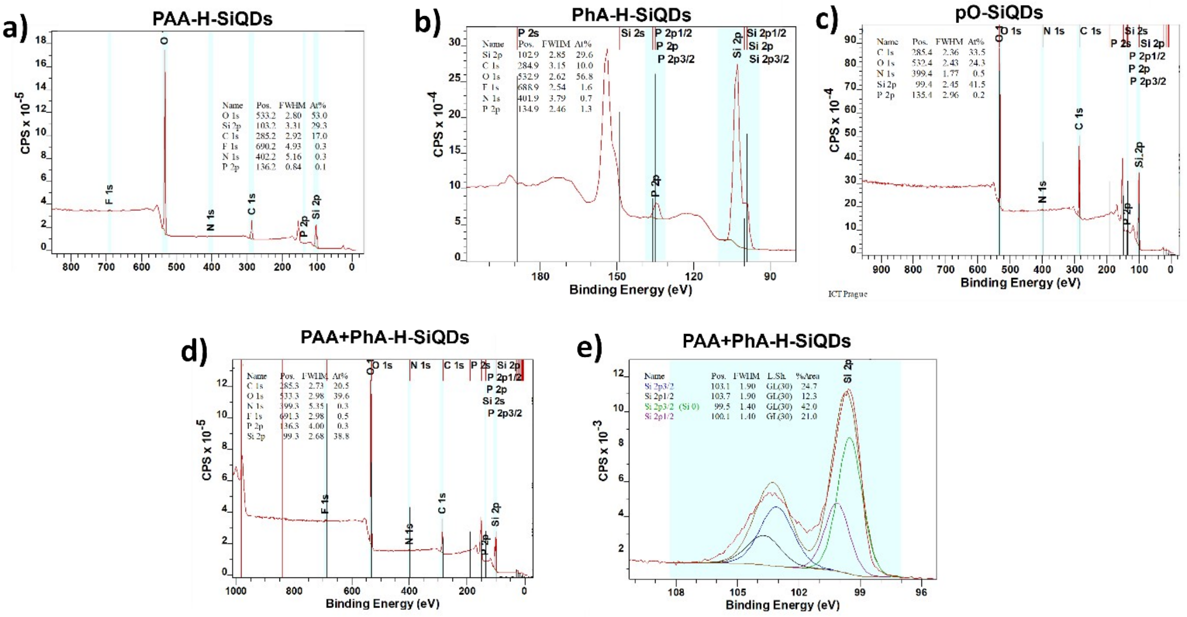

Figure S4. Demonstrative XPS spectra of sample a) PAA-H-SiQDs, b) PhA-H-SiQDs, c) pO-SiQDs and d) PAA+PhA-H-SiQDs. e) Presents examples of Si2p band deconvolution to show the evaluation of concentration of Si^{0+} and Si^{4+}.

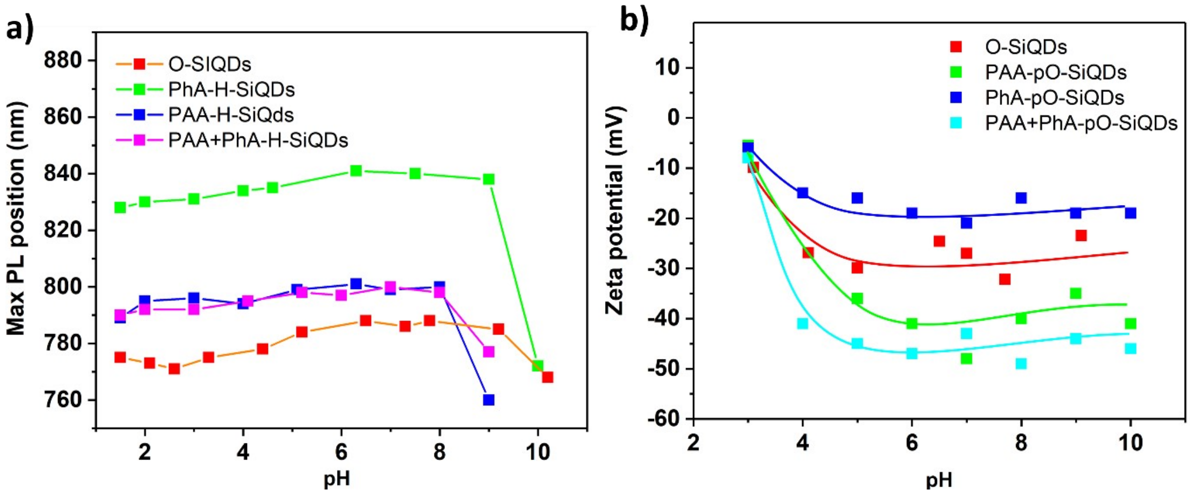

Figure S5. a) Dependence of maxima of PL spectra of samples from H-SiQDs series on pH. b) Zeta-potential dependence on pH of pO-SiQDs series dispersed in water. The lines in a) and b) are only meant to guide the eye.

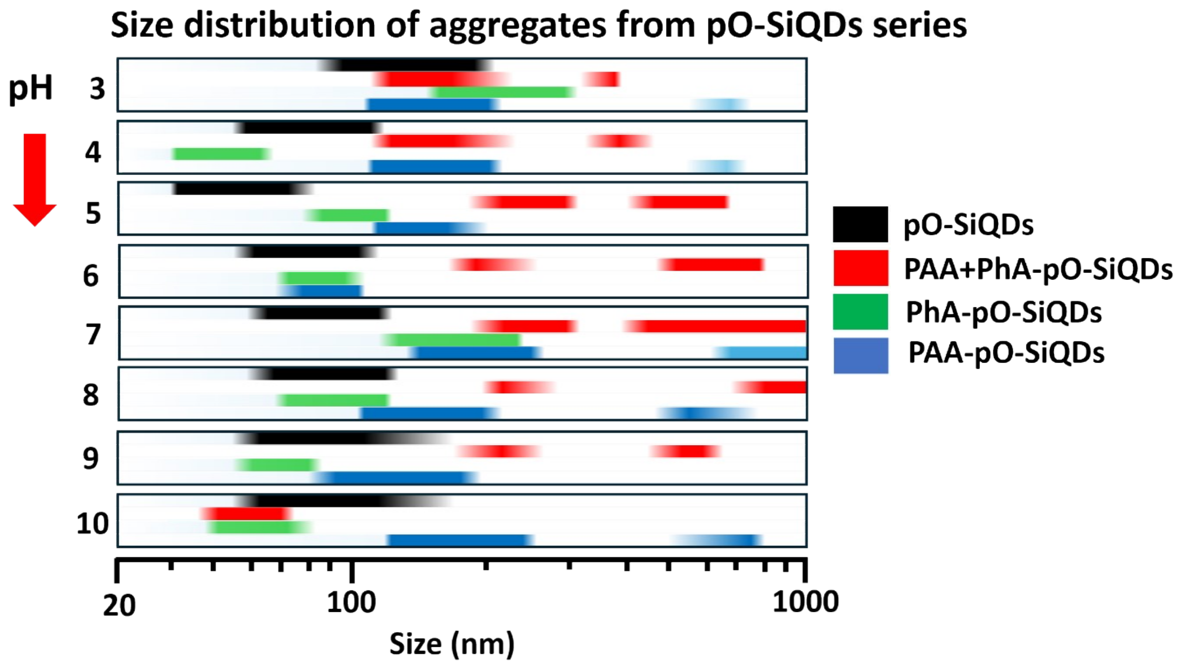

Figure S6. Detailed size distribution of the samples from pO-SiQDs series (Figure 2e). The position and width of each bar reflect the corresponding size distribution curve obtained by DLS measurements.

Table S1: Textural properties of all SiQDs powder measured at 77 K.

Material	S_{BET} (m^2/g)	Total V_{pore} (cm^3/g)	Pore diameter (nm)
**$SiQD_6$**	509.4	1.2464	9.8

References

1. D. König, *AIP Adv.*, 2016, **6**.
2. İ. Doğan, R. Gresback, T. Nozaki and M. C. M. van de Sanden, *Sci. Rep.*, 2016, **6**, 29508.
3. F. Xu, B. Wagner, P. Ghildiyal, L. Mangolini and M. R. Zachariah, *Physical Review Materials*, 2023, **7**, 045403.
